# Correction: Dulski et al. An Organic–Inorganic Hybrid Nanocomposite as a Potential New Biological Agent. *Nanomaterials* 2020, *10*, 2551

**DOI:** 10.3390/nano14201626

**Published:** 2024-10-11

**Authors:** Mateusz Dulski, Katarzyna Malarz, Michał Kuczak, Karolina Dudek, Krzysztof Matus, Sławomir Sułowicz, Anna Mrozek-Wilczkiewicz, Anna Nowak

**Affiliations:** 1 Institute of Materials Engineering, University of Silesia, 75 Pulku Piechoty 1a, 41-500 Chorzow, Poland; 2 Silesian Center for Education and Interdisciplinary Research, 75 Pulku Piechoty 1a, 41-500 Chorzow, Poland; katarzyna.malarz@us.edu.pl (K.M.); mkuczak@us.edu.pl (M.K.); anna.mrozek-wilczkiewicz@us.edu.pl (A.M.-W.); 3 A. Chełkowski Institute of Physics, University of Silesia, 75 Pulku Piechoty 1, 41-500 Chorzow, Poland; 4 Institute of Chemistry, University of Silesia, Szkolna 9, 40-007 Katowice, Poland; 5 Łukasiewicz Research Network - Institute of Ceramics and Building Materials, Refractory Materials Division in Gliwice, Toszecka 99, 44-100 Gliwice, Poland; karolina.dudek@icimb.lukasiewicz.gov.pl; 6 Materials Research Laboratory, Silesian University of Technology, Konarskiego 18a, 44-100 Gliwice, Poland; krzysztof.matus@polsl.pl; 7 Institute of Biology, Biotechnology and Environmental Protection, University of Silesia, Jagiellonska 28, 40-032 Katowice, Poland; slawomir.sulowicz@us.edu.pl; 8 Institute of Nuclear Physics Polish Academy of Sciences, PL-31342 Krakow, Poland; ana.maria.nowak@gmail.com

## Error in Figure

In the original publication [1], there was a mistake in Figure 9, as published. The authors identified an error in the incorrect description of the y-axes’ titles. Specifically, in the lower panel of Figure 9b, the axis title “Cell cycle stage [%]” was corrected to “Cellular subpopulations [%]”. Similarly, in Figure 9c, the title “Cell cycle stage [%]” was corrected to “ROS level [%]”. The corrected Figure 9 appears below. The authors state that the scientific conclusions are unaffected. This correction was approved by the Academic Editor. The original publication has also been updated.

**Figure 9:**
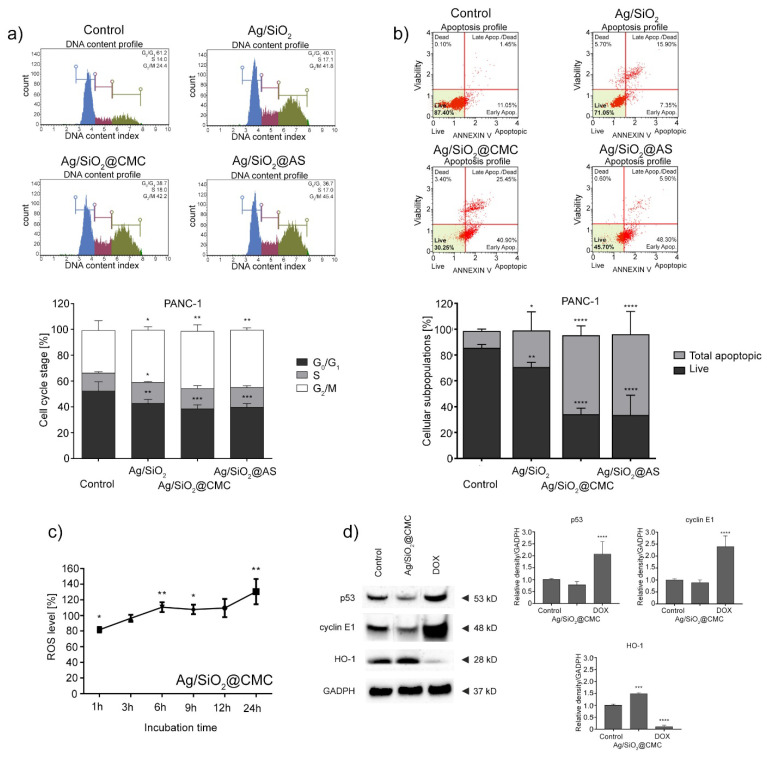
Impact of the tested nanocomposites at a 30 mg/L concentration on regulating the cell cycle (**a**) and inducing apoptosis (**b**) in the PANC-1 cells. Effect of the tested Ag/SiO_2_@CMC nanocomposite on the level of reactive oxygen species (ROS) in the PANC-1 cells. Data normalized to the untreated cells (control) (**c**). Impact of Ag/SiO_2_@CMC on the expression of the p53, cyclin E1, and HO-1 proteins in the PANC-1 cells. The densitometric analysis of these proteins was normalized to GADPH (**d**). The results from all experiments are shown as the mean ± standard deviation (SD) of three independent measurements. Any statistical differences from the cell cycle, apoptosis, and immunoblotting experiments were analyzed using a one-way ANOVA with Bonferroni’s *post-hoc* test. Data from ROS measurements were analyzed using the Student’s *t*-test. Statistical significance: * *p* < 0.05, ** *p* < 0.01, *** *p* < 0.001, **** *p* < 0.0001 compared to the control group.
